# Optimizing a Male Reproductive Aging Mouse Model by d-Galactose Injection

**DOI:** 10.3390/ijms17010098

**Published:** 2016-01-13

**Authors:** Chun-Hou Liao, Bing-Huei Chen, Han-Sun Chiang, Chiu-Wei Chen, Mei-Feng Chen, Chih-Chun Ke, Ya-Yun Wang, Wei-Ning Lin, Chi-Chung Wang, Ying-Hung Lin

**Affiliations:** 1 PhD Program in Nutrition & Food Science, Fu Jen Catholic University, No. 510, Zhongzheng Road, Xinzhuang District, New Taipei City 242, Taiwan; liaoch22@gmail.com; 2 Division of Urology, Department of Surgery, Cardinal Tien Hospital, No. 362, Zhongzheng Road, Xindian District, New Taipei City 231, Taiwan; 3 School of Medicine, Fu Jen Catholic University, No. 510, Zhongzheng Road, Xinzhuang District, New Taipei City 242, Taiwan; 053824@mail.fju.edu.tw; 4 Department of Food Science, Fu Jen Catholic University, No. 510, Zhongzheng Road, Xinzhuang District, New Taipei City 242, Taiwan; 002622@mail.fju.edu.tw; 5 Graduate Institute of Basic Medicine, Fu Jen Catholic University, No. 510, Zhongzheng Road, Xinzhuang District, New Taipei City 242, Taiwan; okwapnice@hotmail.com (C.-W.C.); 081551@mail.fju.edu.tw (W.-N.L.); 075006@mail.fju.edu.tw (C.-C.W.); 6 Research Center for Emerging Viral Infections, Chang Gung University, No. 259 Wen-Hwa 1st Road, Kwei-Shan Taoyuan 333, Taiwan; mfchen0@gmail.com; 7 Department of Urology, En Chu Kong Hospital, No. 399, Fuxing Road, Sanxia District, New Taipei City 237, Taiwan; koacurtis@gmail.com; 8 Department of Chemistry, Fu Jen Catholic University, No. 510, Zhongzheng Road, Xinzhuang District, New Taipei City 242, Taiwan; vic0009@gmail.com

**Keywords:** aging, male infertility, mouse model

## Abstract

The d-galactose (d-gal)-injected animal model, which is typically established by administering consecutive subcutaneous d-gal injections to animals for approximately six or eight weeks, has been frequently used for aging research. In addition, this animal model has been demonstrated to accelerate aging in the brain, kidneys, liver and blood cells. However, studies on aging in male reproductive organs that have used this animal model remain few. Therefore, the current study aimed to optimize a model of male reproductive aging by administering d-gal injections to male mice and to determine the possible mechanism expediting senescence processes during spermatogenesis. In this study, C57Bl/6 mice were randomized into five groups (each containing 8–10 mice according to the daily intraperitoneal injection of vehicle control or 100 or 200 mg/kg dosages of d-gal for a period of six or eight weeks). First, mice subjected to d-gal injections for six or eight weeks demonstrated considerably decreased superoxide dismutase activity in the serum and testis lysates compared to those in the control group. The lipid peroxidation in testis also increased in the d-gal-injected groups. Furthermore, the d-gal-injected groups exhibited a decreased ratio of testis weight/body weight and sperm count compared to the control group. The percentages of both immotile sperm and abnormal sperm increased considerably in the d-gal-injected groups compared to those of the control group. To determine the genes influenced by the d-gal injection during murine spermatogenesis, a c-DNA microarray was conducted to compare testicular RNA samples between the treated groups and the control group. The d-gal-injected groups exhibited RNA transcripts of nine spermatogenesis-related genes (*Cycl2*, *Hk1*, *Pltp*, *Utp3*, *Cabyr*, *Zpbp2*, *Speer2*, *Csnka2ip* and *Katnb*1) that were up- or down-regulated by at least two-fold compared to the control group. Several of these genes are critical for forming sperm-head morphologies or maintaining nuclear integration (e.g., *cylicin, basic protein of sperm head cytoskeleton 2* (*Cylc2*), *casein kinase 2*, *alpha prime interacting protein* (*Csnka2ip*) and *katanin p80* (*WD40-containing*) *subunit B1* (*Katnb1*)). These results indicate that d-gal-injected mice are suitable for investigating male reproductive aging.

## 1. Introduction

### 1.1. Aging and Male Infertility

The increasing trend of delayed parenthood in developed countries is accompanied by an increase in couples with reduced fertility [1,2]. In approximately 50% of couples with reduced fertility, fertility defects can be traced to male partners [3]. In the past decade, several large-scale clinical studies have indicated that male aging is accompanied by decreased semen quality (e.g., reduced total sperm count, concentration and motility), a decrease in the normal morphological sperm ratio and DNA-fragmented sperm [4,5]. In addition, the major reason for diminished semen quality is the accumulation of reactive oxygen species (ROS) and the loss of telomerase activity that accompanies aging [6,7].

### 1.2. Reactive Oxygen Species (ROS) Generation in Testes

The major roles of mitochondria include generating cellular energy and regulating apoptosis and other cellular processes [8]. Energy production through the electron transport chain is coupled with ROS production [8]. Antioxidant enzymes within mitochondria catalyze the ROS to hydro-oxygen and then to H_2_O [9]. However, mitochondrial dysfunction induces excessive ROS production and disrupts the mitochondrial antioxidant system [10]. ROS accumulation causes DNA damage, lipid peroxidation and malformed folding proteins. A clinical study reported that ROS levels were considerably higher in seminal ejaculates of healthy fertile males older than 40 years [6]. In addition, other studies have reported increased ROS levels in the pathogenesis of male infertility [11,12]. In addition, Desai *et al.* indicated that senescence processes in reproductive cells induced ROS accumulation in sperm mitochondria and Leydig cells, resulting in the disruption of sperm telomere, steroidogenesis in the Leydig cells and the presence of mitochondrial DNA in both cells [7].

### 1.3. Effects of Oral Antioxidants on Male Infertility

Several systematic reviews have indicated that oral antioxidants improve sperm quality (*i.e.*, sperm count, motility and morphology) and pregnancy rates [13]. In addition, the use of oral-antioxidant-related products (e.g., vitamin C, vitamin E, selenium, folate, zine and carnitine) in treating men with infertility has been recommended [13,14]. However, individual studies investigating the therapeutic effect of such products have reported conflicting results [15,16]. Nevertheless, animal studies that have validated the effects of oral antioxidants on male reproductive aging are scant.

### 1.4. Model of Male Reproductive Aging

The most prevalent animal models used for investigating male reproductive aging involve using aging rats and d-galactose (d-gal)-injected rat and mouse models [17,18,19,20]. A previous study reported that aging male rats exhibited reduced serum testosterone levels because of decreased serum luteinizing hormone (LH) levels and repressed activates of antioxidant enzymes in Leydig cells [17,18,21]. Another study on aging rats revealed that genes involved in DNA induction damage and repair pathways were affected in the pachytene spermatocytes [22]. Furthermore, d-gal-induced brain aging models have been established for more than 15 years [20,23]. d-Gal is a reducing sugar normally existing in the body. When the concentration of d-gal exceeds normal levels, it is converted to aldehydes and H_2_O_2_ [20,24]. Animals injected with d-gal exhibit aging-related phenotypes, such as neurological defects, decreased immune responses, reduced antioxidant enzyme activity and increased ROS production [20,23,25]. However, studies on male reproductive aging in d-gal-injected mouse models have been reported only in recent years. Only Ahangarpour *et al.* used a d-gal-injected mouse model and reported decreased sperm counts and increased serum LH levels [19]. Nevertheless, details about d-gal-induced aging effects on semen parameters and possible molecular effects on male sperm cells remain unknown. Therefore, we optimized a model of male reproductive aging involving d-gal-injected mice to thoroughly evaluate the effects of d-gal on male reproductive aging.

## 2. Results and Discussion

### 2.1. Optimizing a Model of Male Reproductive Aging through d-Galactose (d-Gal) Injection

**Figure 1:**
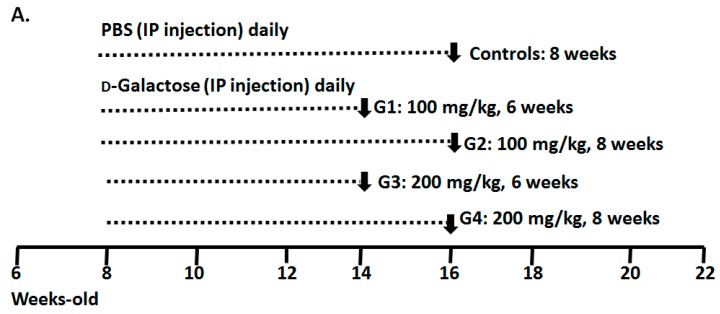

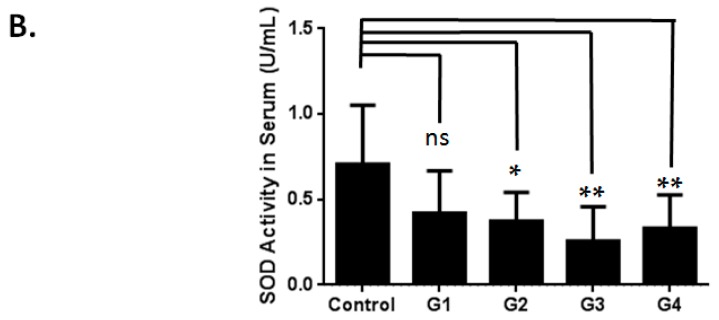
Model of male reproductive aging optimized through d-Galactose (d-Gal) injections. (**A**) Mice divided into five groups: the vehicle group (Control: Phosphate-buffered saline, PBS, administered over eight weeks) and the d-Gal-injected groups, namely G1 (d-Gal dosage of 100 mg/kg over six weeks), G2 (d-Gal dosage of 100 mg/kg over eight weeks), G3 (d-Gal dosage of 200 mg/kg over six weeks) and G4 (d-Gal dosage of 200 mg/kg over eight weeks). The mice were injected intraperitoneally daily for six or eight weeks. The bars indicate the ages of the mice in weeks; (**B**) The serum of mice injected with d-Gal showed lower superoxide dismutase) SOD activity (SOD is a critical antioxidant enzyme) than did the serum of those administered PBS. One-way Analysis of variance (ANOVA) test: “ns” denotes nonsignificant; bars represent the mean ± SD; * *p* < 0.05; ** *p* < 0.01.

**Figure 2:**
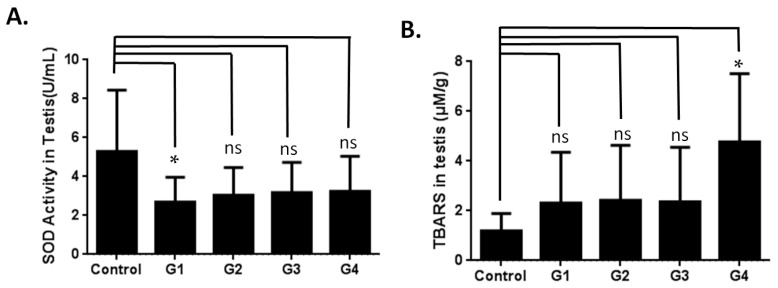
Effects of superoxide dismutase (SOD) activity and thiobarbituric acid reactive substances (TBARS) in testis from mice treated with d-Gal. (**A**) Mice injected with d-Gal l showed decreased SOD activity in testis compared to those administered PBS; (**B**) mice injected with d-Gal showed increased TBARS activity in testis (TBARS is a type of lipid peroxidation) compared to those administered PBS. One-way ANOVA test: bars represent the mean ± SD; Student’s *t*-test: “ns” denotes nonsignificant; * *p* < 0.05.

Previous studies on brain aging have indicated that the recommended d-gal dosage for optimizing an aging model is 50–200 mg/kg over a period of 1–2 months [20,23]. In the current study, to optimize a model of male reproductive aging, a total of 50 mice (aged eight weeks) were divided into five groups according to the administered d-gal dosage and injection period (Figure 1A): Group 1 (G1; a d-gal dosage of 100 mg/kg administered daily over six weeks), Group 2 (G2; a d-gal dosage of 100 mg/kg administered over eight weeks), Group 3 (G3; a d-gal dosage of 200 mg/kg administered over six weeks ), Group 4 (G4; a d-gal dosage of 200 mg/kg administered over eight weeks) and the vehicle group (control group; Phosphate-buffered saline (PBS) administered over eight weeks). The mice were sacrificed within 24 h after the final injection. To determine the effect of d-gal-induced aging, the superoxide dismutase (SOD) activity and the level of an antioxidant maker in the aging model were evaluated in the serum samples. The d-gal-injected groups exhibited significantly lower SOD activity compared to the control group (Figure 1B). In the testicular samples, SOD activity decreased slightly (Figure 2A); compared to the control group, only G1 demonstrated a significant decrease in SOD activity. To thoroughly evaluate the effect of d-gal injection on the aging of the testicular samples, malondialdehyde (MDA), a type of lipid peroxidation, was analyzed using a thiobarbituric acid reactive substances (TBARS) assay. The TBARS levels in the d-gal-injected mice (G1–G4) increased compared to that in the control group (Figure 2B). Among the experimental groups, G4 showed the highest TBARS level. According to the SOD and TBARS levels, antioxidant activity in the d-gal-injected mice decreased, inducing oxidative stress in testis. These results are similar to those obtained in the studies that evaluated brain aging in mouse models [20].

### 2.2. Sperm Quality Decreased in d-Gal-Injected Mice

**Figure 3:**
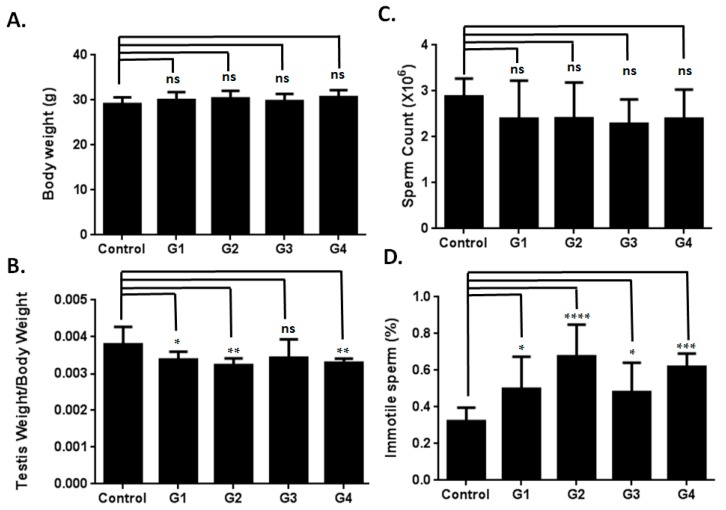
Effects of d-Gal injection on mice (I). (**A**) Body weights from the different groups are similar; (**B**) testis weights/body weights from the d-Gal-injected groups show decreases compared to those of the control group; (**C**) d-Gal-injected groups showed a trend of decreasing sperm counts (×10^6^) compared to the control group; (**D**) the d-Gal-injected mice exhibited an increase in immotile sperm (%) compared to those administered PBS. One-way ANOVA test: bars represent the mean ± SD; “ns” denotes nonsignificant; * *p* < 0.05; ** *p* < 0.01; *** *p* < 0.001; **** *p* < 0.0001.

**Figure 4:**
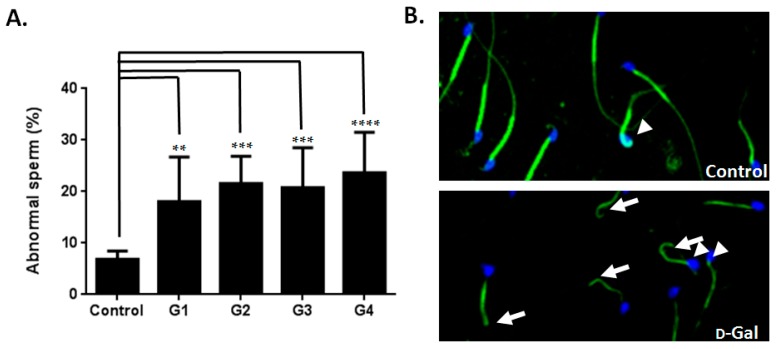
Effects of d-Gal injection on mice (II) (**A**) d-Gal-injected mice showed increased abnormal sperm morphology (%) compared to the control group. One-way ANOVA test: bars represent the mean ± SD; ** *p* < 0.01; *** *p* < 0.001; **** *p* < 0.0001; (**B**) Increased abnormal sperm morphology (%) (arrow: sperm with tail defects; arrowhead: head defects) from mice injected with d-Gal (bottom) compared to those administered PBS. 4,6-diamidino-2-phenylindole (DAPI): blue; Mito Tracker: green (magnification: ×400).

A recent study revealed that testis weight and sperm count were affected in d-gal-injected mice [19]. However, detailed information regarding the effects of d-gal treatment on semen parameters remains lacking. In the current study, the d-gal-injected groups exhibited decreased testis weights compared to the control group (Figure 3A,B). Sperm counts in the d-gal-injected groups demonstrated a decreasing trend compared to those of the control group (Figure 3C). Furthermore, all four experimental groups exhibited a statistically-significant increase in immotile sperm compared to the control group (Figure 3D). All four groups of the d-gal-injected mice also exhibited a statistically-significant increase in abnormal sperm morphology compared to the control group (Figure 4). These results indicated that d-gal treatment induced male reproductive aging.

### 2.3. Investigating the Possible Mechanism of Reproductive Aging in d-Gal-Injected Mice

To identify the affected testicular tissue genes in the d-gal-injected mice, c-DNA microarrays were conducted for a global analysis of gene expression. The microarray analyses indicated that, compared to the control group, the d-gal-injected mice significantly exhibited 129 upregulated genes and 20 downregulated genes (Table S1). We selected *Glutathione peroxidase 3* (*GPx3*), the most upregulated gene, for further evaluation. The total amount of *GPx3* in the d-gal-injected mice was lower than that in the controls (Figure S1). Nine spermatogenesis-related genes were observed (Table 1). Among the spermatogenesis-related genes, eight genes were upregulated (*i.e.*, *Cycl2*, *HK1*, *Pltp*, *Utp3*, *Cabyr*, *Zpbp2*, *Sperr2* and *Csnka2ip*) and one (*i.e.*, *Katnb1*) was downregulated (Table 1). Several of these genes are critical for forming sperm-head morphologies or maintaining nuclear integration (e.g., *cylicin*, the basic protein of sperm head *cytoskeleton 2* (*Cylc2*); *casein kinase 2*, an alpha prime interacting protein (*Csnka2ip*); and *katanin p80* (*WD40* repeat-containing) subunit B1 (*Katnb1*). On the basis of these results, we optimized a model of male reproductive aging and determined the effects of d-gal injection on testicular tissue genes.

**Table 1:** Expressional changes in the spermatogenesis-related genes of the d-gal-injected mice.

Gene	Full Name	Fold
*Cylc2*	Cylicin, basic protein of sperm head cytoskeleton 2	5.62
*Hk1*	Hexokinase 1	2.40
*Pltp*	Phospholipid transfer protein	2.25
*Utp3*	UTP3, small subunit (SSU) processome component	2.20
*Cabyr*	Calcium-binding tyrosine-(Y)-phosphorylation regulated	2.19
*Zpbp2*	Zona pellucida binding protein 2	2.13
*Speer2*	Spermatogenesis associated glutamate (E)-rich protein 2	2.03
*Csnka2ip*	Casein kinase 2, alpha prime interacting protein	2.06
*Katnb1*	Katanin p80 (WD40-containing) subunit B1	0.48

## 3. Materials and Methods

### 3.1. Animal Preparation and Experimental Design

All of the study protocols were executed according to the Guiding Principles for Care and Use of Laboratory Animals and were approved by the Institutional Animal Care and Use Committee (IACUC) of Fu-Jen Catholic University (Identification Code: A10122; 31 July 2012). After an initial acclimatization of 1 week, C57Bl/6 mice were randomized into five groups (each containing 8–10 mice) according to the daily intraperitoneal injection of PBS or 100 or 200 mg/kg dosages of d-gal for 6 or 8 weeks (Figure 1A). The mice were sacrificed 24 h after the final injection under deep anesthesia, and samples (e.g., blood, testes and sperm from the vas deferens) were collected.

### 3.2. Evaluation of Superoxide Dismutase Activity

Superoxide dismutases (SODs) are critical metalloenzymes that catalyze a superoxide anion to H_2_O_2_ [26]. Blood (0.5 mL) and testicular tissue from the mice were injected with vehicle or d-gal. For the serum samples, the blood collected from the treated mice was allowed to clot for 30 min at 25 °C and centrifuged at 2000× *g* for 15 min at 4 °C. Subsequently, the white buffy layer was isolated for further study. For the testis samples, the testes isolated from the mice were homogenized in HEPES (4-(2-hydroxyethyl)-1-piperazineethanesulfonic acid) buffer and centrifuged at 1500× *g* for 5 min at 4 °C. Subsequently, the supernatant was removed. The assay protocol was executed according to the Superoxide Dismutase Assay Kit handbook (No. 706002; Cayman Chemical, Ann Arbor, MI, USA). The final results are presented as U/mg proteins.

### 3.3. Thiobarbituric Acid Reactive Substances (TBARS) Analysis

MDA is a natural product of lipid peroxidation [27]. The MDA level in the testicular tissue was estimated using the TBARS assay [27]. The assay protocol was executed according to the TBARS Assay Kit handbook (No. 10009055; Cayman Chemical, Ann Arbor, MI, USA). The final results are presented as μmole MDA/g proteins.

### 3.4. Semen Parameter

Spermatozoa were collected from the vas deferens of the mice and suspended in a human tubal fluid (HTF) medium (Irvine Scientific, Santa Ana, CA, USA). Regarding sperm counts, sperm cells were immobilized by dilution in water and counted in duplicate by using a hemocytometer (Sigma-Aldrich, Saint Louis, MO, USA). For determining the percent motility, the sperm medium was diluted to 10^6^/mL by using HTF and spotted onto a glass slide. A total of 200 sperm cells (both motile and immotile) were counted in duplicate under a microscope to derive an average percent motility. To analyze the sperm morphology, 100 sperm cells were evaluated on average. Individual sperm cells were categorized as having normal or abnormal morphology (including head, neck and tail defects and immaturity) according to the World Health Organization criteria [3]. The midpiece of each sperm was stained using Mito Tracker conjugated with Alexa Fluor 488 (10 mg/mL) (Invitrogen, Carlsbad, CA, USA); 4,6-diamidino-2-phenylindole (DAPI) was used for nuclear staining.

### 3.5. Microarray Analysis

The murine testicular tissue samples were stored in liquid nitrogen until use by using 2-methylbutane as a cryoprotectant. The total cellular RNA was extracted from the biopsies by using the RNAasey Mini Kit (QIAGEN, Venlo, The Netherlands) according to the manufacturer’s protocol. Total RNA was quantified by measuring the total absorbance at OD_260_/OD_280_ nm. The quality of total RNA was monitored using an Agilent 2100 Bioanalyzer (Agilent Technologies, Waldbronn, Germany). Mouse OneArray^®^ v2 (PhalanxBio, Hsinchu, Taiwan) was used in this assay.

## 4. Conclusions

In this study, we optimized a model of male reproductive aging through d-gal injection and examined its suitability for investigating male reproductive aging. In addition, by conducting cDNA microarray analyses, we identified several spermatogenic genes affected by reproductive aging. These results reveal the possible mechanisms of male reproductive aging.

### 4.1. Effect of d-Gal Injection on the Expression Levels of Spermatogenesis-Related Genes in Mice

In this study, we observed a decreased sperm count and increased ratio of immotile and abnormal sperm morphology. In addition, the microarray analyses reveal that nine spermatogenesis-related genes were affected by d-gal injection. Unexpectedly, the upregulation of the *Cycl2* transcripts of the d-gal-injected mice was five-fold higher than that of the controls. *Cycl2*/*CYCL2* is specifically expressed in the testes and is a part of the basic protein of mammalian sperm heads [28,29]. Moreover, *CYCL2* plays a role in the morphogenesis of sperm heads. The *Katnb1* transcripts of the d-gal-injected mice decreased two-fold compared to those of the controls. Katanin is a heterodimer comprising a 60-kDa ATPase subunit A 1 (*Katna1*) and an 80-kDa accessory protein subunit B 1 (*Katnb1*) [30]. The p60 subunit disassembles microtubules, and the p80 subunit targets katanin to centrosomes. *Katnb1* is presented during spermatid development and is particularly localized in the microtubules of the manchette, a structure required for sperm-head shaping [31]. Mutation in *Katnb1* results in male sterility, which is characterized by decreased sperm count and an absence of progressive sperm in addition to abnormal sperm-head morphology. Genetic alternations of *Katnb1* were observed in infertile men with oligoasthenoteratozoospermia [32]. The spermatogenic genes affected by d-gal injection may reflect the mechanism of spermatogenesis; this phenomenon is similar to the aging process during spermatogenesis.

### 4.2. Model of Male Reproductive Aging

**Figure 5:**
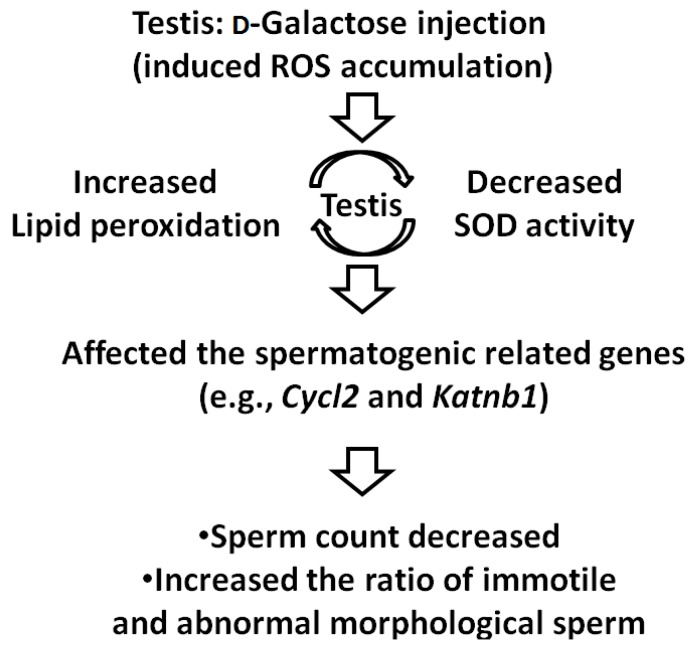
Mouse model of male reproductive aging induced by d-Galactose injection. The d-Gal-injected mice demonstrated decreased SOD activity and increased lipid peroxidation amount. Further, SOD accumulation affects the expression of spermatogenic genes (e.g., *Cycl2* and *Katnb1*) and results in the decreased sperm count and increased ratio of immotile and abnormal sperm morphology.

A previous study reported that d-gal forms advanced glycation end products (AGEs) *in vivo* and that elevated levels of AGEs may accelerate the aging process [20]. In our study, the d-gal-injected mice demonstrated decreased SOD activity and increased lipid peroxidation levels. These results are consistent with those of previous studies that have applied d-gal to induce brain aging in mice (Figure 5) [23,25,33]. Low SOD activity reflects normal male aging [34]. Previous studies involving d-gal-injected rats have reported that the rats exhibited a decrease in sperm count and a regression of the testes, which is consistent with our results [34]. However, only one study that employed d-gal-injected mice for evaluating male reproductive aging revealed decreased sperm counts and increased serum LH levels [19]; our results, which showed decreased testis weights and lower sperm counts, are consistent with the results of this study. Furthermore, we found an increased ratio of immotile sperm and abnormal sperm morphology in the d-gal-treated mice (Figure 5); this finding is highly consistent with the typical characteristics of aging males (e.g., reduced total sperm count, concentration and motility; increased abnormal sperm morphology) [4,5]. On the basis of these results, we conclude that the optimized model of reproductive aging involving d-gal-injected mice is suitable for investigating male reproductive aging. Although previous studies have recommended using antioxidants for treating infertility involving ROS in males, the reports of studies on the therapeutic effects of such antioxidants have been contradictory [13,15,16]. Therefore, we optimized this model for evaluating the effects of antioxidants on male reproductive aging.

### 4.3. Conclusions

d-gal injection induced male reproductive aging and may affect several spermatogenic genes. d-gal-treated mice may be excellent candidates for demonstrating the aging of the male reproductive system.

## Supplementary Materials

Supplementary materials can be found at http://www.mdpi.com/1422-0067/17/1/98/s1.
