# Supplementary material for: Optimizing a Male Reproductive Aging Mouse Model by d-Galactose Injection
Source: Int J Mol Sci. 2016 Jan 13;17(1):98. doi: 10.3390/ijms17010098 (PMC4730340; doi:10.3390/ijms17010098)
Supplement: Supplementary file 1 [file ijms-17-00098-s001.pdf]

Supplementary Information: Optimizing a Male Reproductive Aging Mouse Model by D-Galactose Injection

Chun-Hou Liao, Bing-Huei Chen, Han-Sun Chiang, Chiu-Wei Chen, Mei-Feng Chen, Chih-Chun Ke, Ya-Yun Wang, Wei-Ning Lin, Chi-Chung Wang and Ying-Hung Lin

Table S1. List of expressional changes of up- regulated and down-regulated genes from D-gal-injected mice.

Gene	Full Name	Fold
	Up-regulated genes	
Gpx3	Glutathione peroxidase 3	13.1
C3	Complement component 3	8.5
H2-Aa	Histocompatibility 2, class II antigen A, α	6.9
Ctss	Cathepsin S	6.3
Cylc2	Cylicin, basic protein of sperm head cytoskeleton 2	5.6
H2-Eb1	Histocompatibility 2, class II antigen E β	5.2
Psmb7	Proteasome (prosome, macropain) subunit, β type 7	4.3
Frg1	FSHD region gene 1	4.2
Safb2	Scaffold attachment factor B2	4.1
Gbp2	Guanylate binding protein 2	4
B2m	β-2 microglobulin	4
Tbx3	T-box 3	3.8
Snrpa	Small nuclear ribonucleoprotein polypeptide A	3.7
Smarca2	SWI/SNF related, matrix associated, actin dependent regulator of Chromatin, subfamily a, member 2	3.7
Gm13237	Predicted gene 13237	3.6
Cep85l	Centrosomal protein 85-like	3.5
Dcn	Decorin	3.2
Nme8	NME/NM23 family member 8	3.2
Dhx9	DEAH (Asp-Glu-Ala-His) box polypeptide 9	3.2
Dnajb11	DnaJ (Hsp40) homolog, subfamily B, member 11	3.2
Sltm	SAFB-like, transcription modulator	3.1
Fus	Fused in sarcoma	3.1
Anxa1	Annexin A1	3.1
Ly86	Lymphocyte antigen 86	3
Hpgd	Hydroxyprostaglandin dehydrogenase 15 (NAD)	3
Ccdc112	Coiled-coil domain containing 112	3
Efcab2	EF-hand calcium binding domain 2	3
C1qc	Complement component 1, q subcomponent, C chain	3
Dnajc21	DnaJ (Hsp40) homolog, subfamily C, member 21	2.9
Ccl8	Chemokine (C–C motif) ligand 8	2.9
Ccdc186	Coiled-coil domain containing 186	2.9
Plagl1	Pleomorphic adenoma gene-like 1	2.8
Amy1	Amylase 1, salivary	2.8
Cdkl2	Cyclin-dependent kinase-like 2 (CDC2-related kinase)	2.8
Nucb2	Nucleobindin 2	2.8
Ifitm2	Interferon induced transmembrane protein 2	2.8

Table S1. *Cont.*

Gene	Full Name	Fold
Dock8	Dedicator of cytokinesis 8	2.8
Slc24a5	Solute carrier family 24, member 5	2.7
Fam98b	Family with sequence similarity 98, member B	2.7
Gstm2	Glutathione S-transferase, mu 2	2.7
Stx4a	Syntaxin 4A (placental)	2.7
Ik	IK cytokine	2.7
Mki67	Antigen identified by monoclonal antibody Ki 67	2.7
Nsmce2	Non-SMC element 2 homolog (MMS21, *S. cerevisiae*)	2.6
Efemp1	Epidermal growth factor-containing fibulin-like extracellular Matrix protein 1	2.6
Dnajc2	DnaJ (Hsp40) homolog, subfamily C, member 2	2.6
Snrnp70	Small nuclear ribonucleoprotein 70 (U1)	2.6
Wfdc17	WAP four-disulfide core domain 17	2.5
Hpgd	Hydroxyprostaglandin dehydrogenase 15 (NAD)	2.5
Leo1	Leo1, Paf1/RNA polymerase II complex component, homolog	2.5
Cenpe	Centromere protein E	2.5
Fam71d	Family with sequence similarity 71, member D	2.5
Zmynd11	Zinc finger, MYND domain containing 11	2.5
Zfml	Zinc finger, matrin-like	2.5
Sult1e1	Sulfotransferase family 1E, member 1	2.4
Irf7	Interferon regulatory factor 7	2.4
C1ra|C1rb	Complement component 1, r subcomponent A|complement Component 1, r subcomponent B	2.4
Ccdc104	Coiled-coil domain containing 104	2.4
Wdr66	WD repeat domain 66	2.4
Nae1	NEDD8 activating enzyme E1 subunit 1	2.4
Col4a3bp	Collagen, type IV, α 3 (Goodpasture antigen) binding protein	2.4
Hk1	Hexokinase 1	2.4
Phyh	Phytanoyl-CoA hydroxylase	2.4
Gm136	Predicted gene 136	2.4
Hey1	Hairy/enhancer-of-split related with YRPW motif 1	2.4
Ccdc66	Coiled-coil domain containing 66	2.4
Aldh1a7	Aldehyde dehydrogenase family 1, subfamily A7	2.4
Nemf	Nuclear export mediator factor	2.4
Hsd3b1	Hydroxy-δ-5-steroid dehydrogenase, 3 β- and steroid δ-isomerase 1	2.4
Hirip3	HIRA interacting protein 3	2.3
Ncl	Nucleolin	2.3
Tmem176b	Transmembrane protein 176B	2.3
Wasf2	WAS protein family, member 2	2.3
Fxr1	Fragile X mental retardation gene 1, autosomal homolog	2.3
Gpatch4	G patch domain containing 4	2.3
Gucy1a3	Guanylate cyclase 1, soluble, α 3	2.3
Ino80b	INO80 complex subunit B	2.3
Cdk11b	Cyclin-dependent kinase 11B	2.3
Morf4l2	Mortality factor 4 like 2|mortality factor 4-like protein 2-like	2.3
Serpinf1	Serine (or cysteine) peptidase inhibitor, clade F, member 1	2.3
Top2a	Topoisomerase (DNA) II α	2.3

Table S1. *Cont.*

Gene	Full Name	Fold
Nipbl	Nipped-B homolog (Drosophila)	2.3
Gm4907	Predicted gene 4907	2.3
Ankrd36	Ankyrin repeat domain 36	2.3
Pltp	Phospholipid transfer protein	2.2
Igf2bp2	Insulin-like growth factor 2 mRNA binding protein 2	2.2
Senp5	SUMO/sentrin specific peptidase 5	2.2
Plcb1	Phospholipase C, β 1	2.2
Dcn	Decorin	2.2
Sumo1	Small ubiquitin-like modifer 1	2.2
Dnttip2	Deoxynucleotidyltransferase, terminal, interacting protein 2	2.2
Zrsr1	Zinc finger (CCCH type), RNA binding motif and serine/arginine rich 1	2.2
Utp3	UTP3, small subunit (SSU) processome component, homolog (*S. cerevisiae*)	2.2
Cabyr	Calcium-binding tyrosine-(Y)-phosphorylation regulated (fibrousheathin 2)	2.2
Anp32e	Acidic (leucine-rich) nuclear phosphoprotein 32 family, member E	2.2
Pinx1	PIN2/TERF1 interacting, telomerase inhibitor 1	2.2
Epsti1	Epithelial stromal interaction 1 (breast)	2.2
Nop56	NOP56 ribonucleoprotein	2.2
Ndc80	NDC80 homolog, kinetochore complex component (*S. cerevisiae*)	2.2
Smim24	Small integral membrane protein 24	2.2
Ccdc173	Coiled-coil domain containing 173	2.2
Hnrnpul2	Heterogeneous nuclear ribonucleoprotein U-like 2	2.2
Acsl4	Acyl-CoA synthetase long-chain family member 4	2.1
Aggf1	Angiogenic factor with G patch and FHA domains 1	2.1
Zpbp2	Zona pellucida binding protein 2	2.1
F13a1	Coagulation factor XIII, A1 subunit	2.1
Atg3	Autophagy related 3	2.1
Fhad1	Forkhead-associated (FHA) phosphopeptide binding domain 1	2.1
Tns1	Tensin 1	2.1
Senp2	SUMO/sentrin specific peptidase 2	2.1
Mapk1ip1	Mitogen-activated protein kinase 1 interacting protein 1	2.1
Grk1	G protein-coupled receptor kinase 1	2.1
Wdr43	WD repeat domain 43	2.1
Kank2	KN motif and ankyrin repeat domains 2	2.1
Rrbp1	Ribosome binding protein 1	2.1
Terf1	Telomeric repeat binding factor 1	2.1
Rdx	Radixin	2.1
Sult1a1	Sulfotransferase family 1A, phenol-preferring, member 1	2.1
Pdgfra	Platelet derived growth factor receptor, α polypeptide	2.1
Csnka2ip	Casein kinase 2, α prime interacting protein	2.1
Snrnp48	Small nuclear ribonucleoprotein 48 (U11/U12)	2.1
Wdr26	WD repeat domain 26	2
Tmco5	Transmembrane and coiled-coil domains 5	2
Suco	SUN domain containing ossification factor	2
Polr3gl	Polymerase (RNA) III (DNA directed) polypeptide G like	2
Ddr2	Discoidin domain receptor family, member 2	2

Table S1. *Cont.*

Gene	Full Name	Fold
Speer2	Spermatogenesis associated glutamate (E)-rich protein 2	2
Rwdd1	RWD domain containing 1	2
Tshz2	Teashirt zinc finger family member 2	2
	Down-regulated genes	
Gene_symbol	Description	Fold
Rn28s1	28S ribosomal RNA	0.28
Snord13	Small nucleolar RNA, C/D box 13	0.32
Snord118	Small nucleolar RNA, C/D box 118	0.36
2900060B14Rik	RIKEN cDNA 2900060B14 gene	0.37
Rs5-8s1	5.8S ribosomal RNA	0.37
Rn18s	18S ribosomal RNA	0.38
Rn7sk	RNA, 7SK, nuclear	0.41
Cyp4f39	Cytochrome P450, family 4, subfamily f, polypeptide 39	0.45
Lbr	Lamin B receptor	0.45
Comp	Cartilage oligomeric matrix protein	0.46
Npas1	Neuronal PAS domain protein 1	0.46
Pomc	Pro-opiomelanocortin-α	0.47
Pfn1	Profilin 1	0.47
Zdhhc1	Zinc finger, DHHC domain containing 1	0.48
Lrrc29	Leucine rich repeat containing 29	0.48
Katnb1	Katanin p80 (WD40-containing) subunit B 1	0.48
Nek8	NIMA (never in mitosis gene a)-related expressed kinase 8	0.49
Mau2	MAU2 chromatid cohesion factor homolog (*C. elegans*)	0.49
Sec1	Secretory blood group 1	0.49
Cnnm4	Cyclin M4	0.49

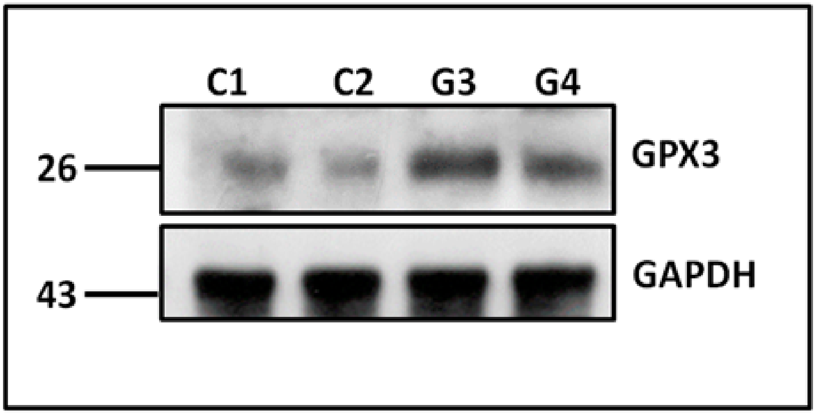

Figure S1. The expressional levels of GPX3 in testis from D-gal injection and control mice. Western blot analysis of testis by D-galactose injection (G3 and G4) and controls (C1 and C2) using an anti-GPX3 and an anti-GAPDH antibody.
